# Where Are the Months? Mental Images of Circular Time in a Large Online Sample

**DOI:** 10.3389/fpsyg.2019.02634

**Published:** 2019-11-28

**Authors:** Bruno Laeng, Anders Hofseth

**Affiliations:** ^1^Department of Psychology, University of Oslo, Oslo, Norway; ^2^RITMO Centre for Interdisciplinary Studies in Rhythm, Time and Motion, University of Oslo, Oslo, Norway; ^3^NRKbeta, The Norwegian Broadcasting Corporation, Oslo, Norway

**Keywords:** time, imagery, mental models, spatialization, motion, synesthesia

## Abstract

People may think about time by mentally imaging it in some spatial form, or as “spacetime.” In an online survey, 76,922 Norwegian individuals positioned two dots corresponding to the months of December and March on what they imagined to be their appropriate places on a circle. The majority of respondents placed December within a section of the circumference ranging from 11:00 to 12:00 o’clock, but a group of respondents chose positions around the diametrically opposite 6:00 o’clock position. A similar relationship occurred for March, where most respondents chose a position ranging from 2:30 to 3:00 o’clock but a group of respondents chose positions around 9:00 o’clock. About half of the respondents (*N* = 39,797) continued to fill out an online questionnaire probing their mental images related to the “year” concept. This clarified that 75% of respondents “saw” the months unfolding in a clockwise direction versus 19% in a counter clockwise fashion. Moreover, while a majority (70%) stated that they imagined the year as a “circle,” the rest indicated the use of other mental images (e.g., ellipses and spirals, lines and squares, idiosyncratic or synesthetic spatial forms). We found only weak effects or preferences for spatial forms based on respondents’ gender, handedness, age, or geographical location.

Eternity is like unto a Ring.

Time, like to Measure, doth it self extend;

Measure commences, is a finite thing.

The Ring has no beginning, middle, end.

–(John Bunyan, 1628–1688)

## Introduction

Recently, one of us witnessed a bizarre discussion: a colleague had been assigned to designing a time planning tool called a *year wheel* for the office’s activities. Everybody seemed to have differing opinions on *where* the different months should be located on the wheel, so the colleague tried to settle it by posting a piece of paper with a circle and a simple question: “If the year is a circle – where’s March and December in your mind?” The results seemed to confirm that many people find it fairly simple to visualize a circular spatial form for a longer time period like a year, but opinions on *where* the months belonged, or what way the circular progress of time should go, varied wildly; and opinions were strongly held. The example suggests that many people are able to visualize the past and the future by generating mental images of temporal dimensions construed spatially, and that these mental images can be individual, and not necessarily conformed to a single common model. Unlike things we can see, hear, or touch, time cannot be sensed directly. Hence, spacetime images may serve as useful “mental models” for how something works in the real world. As originally suggested by the psychologist Craik (1943), the human mind tends to construct many “small-scale models” of reality (cf. Johnson-Laird, 1983) that are used to anticipate events and allow the conceiving, reasoning, and mental manipulation of relations (Gottschling, 2006). For time concepts, this is especially valuable, since spatial models usually can be described verbally, and consequently, it is possible to convey meaning to others in speech (Casasanto and Boroditsky, 2008; Bender and Beller, 2014). When spatializing time, either mentally or with “time-reckoning” instruments (Evans, 2013), the distance covered by the clock’s hands turns the immaterial “time” into a material object, which can be indicated, measured, and communicated (Canales, 2015).

In cognitive neuroscience theories of mental imagery (e.g., Kosslyn, 1980, 1994), representations of objects or scenes that are not physically present are mentally re-constructed. These “visual (mental) images” necessarily occur within a “spatial medium.” Specifically, using the topographic layout of the neural retinotopic or spatiotopic maps supporting ordinary perceptions (e.g., Kosslyn et al., 2006), we can mentally simulate the perception of objects and their parts by preserving their spatial relations. Moreover, dynamic mental images that unfold in time in a kinematic sequence (like “movies in the head”) may preserve the movements (Laeng and Teodorescu, 2002; de’Sperati, 2003) and the temporal ordering of events by representing changes in their spatial position or our viewpoint. Indeed, the mental or neural maps of distance and durations may also be defined as ordered and sequential activities of neuronal assemblies (e.g., in the brain’s hippocampus) representing inter-event relationships (Eichenbaum, 2017; Buzsáki and Tingley, 2018).

Not all cultures would seem to define or experience time in the same way (Bender and Beller, 2014; Sinha and Gärdenfors, 2014), and importantly, time is not always spatialized and it can be conceptualized as “event-based” time, without space-time mapping (e.g., Silva Sinha et al., 2012; Silva Sinha, 2019). Although time is often spatialized, specific conceptualizations of time differ and some might favor a linear or “open-ended” nature of time, where time begins at one point and then ends at another (e.g., in Christianity, Islam, and Judaism). In literate cultures, a “mental time line” may be a pervasive cognitive structure (Eikmeier et al., 2013; Weger and Pratt, 2013). Its “arrow of time” seems influenced by the direction of script, sight-reading, or tactile path in Braille (e.g., Tversky et al., 1991; Fuhrman and Boroditsky, 2010; Ulrich and Maienborn, 2010; Santiago et al., 2010; Bergen and Chan Lau, 2012; Bottini et al., 2015; Aguirre and Santiago, 2017; but see also Santiago et al., 2007; Casasanto and Bottini, 2013). In modern western cultures, the spatializing time in general appears gradually during childhood and depends on schooling and education (e.g., Droit-Volet and Coull, 2015; Tillman et al., 2018). However, one can also view the Neolithic circles of stones (e.g., *Stonehenge*) as spatializations of time (Lewis-Williams and Pearce, 2005) expressed independently from literacy in these prehistoric cultures (d’Errico, 1998). All societies, past and present, appear to display multiple concepts of time, not singular, pointing to multiple ways of “perceiving” or “imagining” time according to the context (Lucas, 2005, p. 94). Some societies express temporal structure within an “ordinal” system (Lucas, 2005, p. 94), rather than a regular “interval” system (typical of time-reckoning systems like clocks) in mental timelines and axes (Rodríguez, 2019). Ordinal chronologies express “periods” of non-specific duration, where the passing of time is marked in a relative succession of phases, instead measured in absolute terms. Such an ordinal system, if based on broad classes of natural phenomena such as the astronomical (motions of stars and planets) and seasonal (climate and biological organisms) can include “recursivity” – albeit often with irregularly spaced periodic divisions – and can ultimately be considered cyclical, or as a shape folding back on itself. Some chronologies (e.g., in indigenous Brazilian cultures, Silva Sinha, 2019) can entirely disregard the idea of a linear or cumulative flow of time.

Nature-oriented aspects of time can all be reduced to a single category labeled “eco-time” by the astronomer (Aveni, 2002; see also MacDonald, 2003, p. 93). Eco-time connects people with the environment through their reactions to the rhythms of nature. In addition, regular festivities and celebrations transform eco-time into “socio-time,” a temporal organization of social practice (Silva Sinha, 2019). Through socio-time, people connect to family members (e.g., birthdays, Christmas, etc), society (e.g., holidays, National Day, or Labor Day), the religious community (e.g., Easter, Ramadan or Ashura), or the world at large (e.g., New Year’s Eve). The seasonality of rites and tasks would seem to prime a cyclical spatial model of the “year” in general, which would seem likely to take paramount relevance for every person in a society (North, 2003).

In nature, the apparent circular paths of the astronomical bodies, their stable direction and repetitiveness of motion, the periodicity of the year’s seasonal changes in daylight, and the tides of the oceans could single out the circular shape as the optimal spatial model for the coming and going of time. Indeed, when modern tools for time reckoning emerged, the circular revolutions made by the clock’s hands (or a sundial’s shadow) embodied the astronomical motions and other rhythms of nature. At latitudes above the Polar Circle, one can actually experience the sun completing a full circle above the horizon in the span of a day (e.g., in Northern Norway; but only during the midsummer or “midnight sun” period; Laeng et al., 2007). Nowadays, although there are languages that systematically integrate gestures toward the sky to speech to narrow down temporal reference (e.g., pointing along the east-west axis of the sun’s arc for time-of-day reference; Floyd, 2016), the great majority of humans do not typically look at the sun or the stars to keep track of time or to communicate explicitly about it. Indeed, the planet Earth is currently populated by “clocked cultures” (Eco, 2003; Williams, 2017), where mechanical and digital timekeepers are ubiquitous and have a much greater precision than previous means of measuring time.

Clocks and calendars serve as “cognitive artifacts” (Sinha et al., 2011; Silva Sinha et al., 2012), “material anchors” (Hutchins, 2005), “cultural constructions” (Gell, 1992), or “extended cognition” (Clark and Chalmers, 1998), allowing the projection of spatial concepts into a “conceptual blend” (Fauconnier and Turner, 2003) from which a specific concept of time takes shape. The classic “clock face” has a standard spatial structure imitating the older sundial, parsing and ordering the hours, but completing the sundial’s cycle as a full circle, where time is traveling by night as well. In other words, clocks seem to serve a double cognitive function by both measuring time and symbolizing it as a continuous and cyclical spatial dimension (Duffy, 2014).

In principle, as long as time is moving in a curvilinear fashion, it is not relevant in which particular direction the motion takes place. On a sundial, the daily sunlight will cause a casted shadow line, fully visible during daylight, and moving “clockwise” (by aiming the dial to the north in the Northern hemisphere) from the early to late daytime hours. The classic clock’s hands movements appears to re-enact the sundial’s shadow motions in another key dimension: the direction of movement of time. An additional human factor that could bias the circular spatial model of time toward a specific direction of motion is manual *torque* or the angular force required to act on something. Humans have a general preference for manual action in one direction of torque since a great majority of people are right-handed, and supination (a movement away from the body) of the arm is stronger than pronation (a movement toward the body). Since the right arm is stronger in most people (Peters and Servos, 1989), most artifacts require a clockwise action when holding and turning, tightening something (e.g., a light bulb, screws, bolts, caps, and lids; see Coren, 1993, pp. 240–241; McManus, 2002, p. 46). Note that the opposite counter-clockwise action is associated with the opposite action, e.g. opening or releasing. It remains unclear whether the image of a clockwise motion may be evoked also when the motion is metaphorical, and no action is literally required, as one could possibly posit on the basis of models of cognitive “force dynamics” (Talmy, 1988) and “embodied” cognitive compatibility effects (e.g., Glenberg and Kaschak, 2002; Lugli et al., 2018). In these cases, meaning derives from the biomechanical nature of bodies and perceptual systems (Lakoff, 1987; Glenberg, 1997). Thus, in an “embodied simulation” account, a clockwise movement could match the abstract concept of the “closing” of an event, whereas the opposite concept of “opening” would match a counter clockwise motion.

There is some evidence for a preference in the general population for “mentally rotating” abstract shapes clockwise in one’s head (e.g., Koriat and Norman, 1985; Liesefeld and Zimmer, 2011). Martin and Jones (1999) suggested the presence of a general handedness effect on cognition (referred as “chiral” psychology) that may reflect specific patterns of underlying motor activation. In sports that occur within a circular path or ring (e.g., running, skating), the direction of motion is rigorously anti-clockwise. This may reflect that the right side of the human body (e.g., the right leg) tends to be stronger in a majority of the population, leading to stronger propelling thrusts in a counter-clockwise than clockwise direction. It has apparently also influenced horse races, car, and cycle races, which follow this counterclockwise principle.

Our theoretical stance is that any person’s idea of time tends to be composed of multiple concepts, with context determining which one is active. Thus, someone’s circular spatial model of the year may not be exactly analogous to the fixed layout and motion of wristwatches and clocks.

In the present study, we examine a host of possible factors (e.g., handedness, gender, age, and geographical location) that may influence a person’s mental image of time. Regarding *handedness*, for instance, would right-handers show a stronger bias for a clockwise year than left-handers? Does age play a role? We could surmise that as older people become more aware of physical decay, or that time will end (Yamada and Kato, 2006), they could imagine a unidirectional timeline, literally terminating at a “deadline.” Alternatively, older individuals may be more aware of the “life cycle” by experiencing the re-occurrence of developmental changes or “stages of life” (Hendry, 2003) in the younger generations. In either case, growing old could results in a modulatory effect away from the typical bias of younger individuals. Finally, given that most of our respondents grew up in Norway, we assessed whether those from the northernmost regions (i.e., the Arctic region) have a stronger preference for a circular spatial model of time; for instance based on experiencing a complete circular course of the sun in the sky in the summer months.

At the psychological or individual level, it may seem natural that spatial externalizations can switch between linear and circular forms, depending on the imagined timeframe, each adapted to express two temporal phenomena: repetitions in nature and society, versus the irreversibility of linear decay. The circular spatial model seems pervasive for representing the sequence and perhaps the subjective “duration” of time, of the day or year, since several salient events in nature seem isomorphic to this specific model (e.g., the regular rising and setting of the sun and other stars, the waxing and waning of the moon). Indeed, some modern calendars can take a circular form (e.g., the so-called “planning year wheel” of offices or schools). The most common graphical organization for calendars seems however to be the matrix (e.g., a 4/5 × 7 array, with days as rows, and weeks as columns, split by months, or a 4 × 3 matrix of months; Duffy, 2014). Such a square-like type of spatial layout seems highly practical. Indeed, there is evidence that linear rows or rectangles may be the default image for the months of the year in many individuals (but not for the “calendrical” synesthetes; Brang et al., 2010). In particular, a frame of reference at the basis of much linguistic communications is the so-called “mental time line,” which is an imaginary sequence framework extending in space (Bender et al., 2012; Eikmeier et al., 2013; Weger and Pratt, 2013; Bender and Beller, 2014). Such a mental time line has a direction that is approximately rectilinear or curvilinear and may be irreversible in motion. Yet, the mental time line may be also reversible, as a curved line with time curving as a pendulum, or according to a specific trajectory that however joins ends in a full circle (Leone et al., 2018).

Thus, in the present study, we invited Norwegian web readers to consider the possibility that a year could be a circle by positioning 2 months (i.e., December and March) on an abstract circle, displayed on screen, by clicking their imagined positions along the circumference. Subsequently, we requested them to continue to fill out a questionnaire, which also probed the degree to which our respondents would actually subscribe to a circular mental image, instead of simply playing along the “what-if” requirement of our test, and if other spatial images would actually be preferred. The survey allows us to document a phenomenon that had perhaps first received attention by Galton (1907) in his empirical work on “number forms” based on collecting sketches, drawings, or arrangements of tokens, from a variety of individuals. Galton anticipated several key ideas of modern theories of mental imagery (e.g., Laeng and Sulutvedt, 2014), initiated the empirical study of synesthetic experiences (like the so-called “number forms”; Galton, 1881; Seron et al., 1992; Sagiv et al., 2006), and devised the questionnaire method (Kindley, 2016, p. 9). All of the above are relevant elements of the present study.

A notable example of applying Galton’s method, is a study by Leone et al. (2018) where participants are requested to position on a blank computer screen a finite number of geometrical shapes (either circles or rectangles) representing each of the seasons. Statistical analyses revealed an average bias of the group of respondents to position the four seasons in an approximately circular arrangement and according to a counterclockwise arrangement. Interestingly, when arranging the days of the week using the same method, the arrangement appeared to be linear (progressing from left to right and gradually upward). In addition, in one condition, the participants initially saw a circle on screen and they partitioned it in variably sized wedges, corresponding to the different parts of the day (i.e., morning, afternoon, and night). About one-third of the participants followed a counterclockwise arrangement of the parts of the day, indicating that the clock’s hands’ direction of movement is not necessarily a spatial model informing every individual’s image of cyclical time progression.

The study’s approach of “prompting” respondents to adopt a circular spatial frame from the start may differ from many tasks used previously in the literature. We believe that the method can complement the open-ended approach by Galton. Although the form generation method seems ideal for revealing the prevalence of a specific spatial shape and the potential to reveal the varieties of forms in the population, Galton-like methods leave the important question unanswered of whether an individual’s particular form of spatialization is unique and fixed, or flexible and available as an alternative representation. Hence, prompting people to take a specific perspective or adopt a circular spatial frame, even among individuals that have not thought about the issue in such terms, can throw light on whether a specific spatial model can be adopted when thinking about time. Moreover, we cane estimate its ease by probing with rating scales its naturalness.

## Methods

### The Online Survey

We first run an Internet-based survey in Norwegian at the website of the major, state-owned, Public Service Broadcaster of the country (NRK: *Norsk rikskringkasting*; tr. *Norwegian National Broadcasting* or *Norwegian Broadcasting Corporation*). This online survey was able to successfully collect responses from a large sample of Norwegian participants, which to our knowledge is the largest sample ever collected on this topic (e.g., a previous large online survey had *N* = 1,454; Leone et al., 2018).

### Respondents

A total of 76,922 individuals responded to the first interactive item of the survey. They positioned a month label on what they thought would be its appropriate place on the circumference of an empty circle. A substantial group (*N* = 39,797) went on to respond to a more detailed questionnaire. All respondents voluntarily answered an identical series of simple questions prompting respondents to “introspect” their own mental images. Participation in the survey was voluntary. Respondents were anonymous and their actual location, IP number, or other identifying information (e.g., name) were not registered; however, they could get in touch with A.H. *via* the form or by email if they wanted more information about the survey. We removed obvious duplicates (identical answers across all parameters entered within a short time-frame) from the dataset. We conducted the whole data collection in the *NRKbeta* subsection of the general media platform NRK.no, containing information, news, and entertainment from the Norwegian Broadcasting Company. The article gathering the data was displayed to 152,000 unique visitors during the data gathering period, with 71% of traffic originating from the front page of NRK.no, which is exposed to a fairly large and wide cross-section of the Norwegian population, 10% coming from Facebook, and most of the rest (14%) coming from people arriving directly to NRKbeta. This implies a response rate of 50% for the circle input and 26% for the questionnaire.

### Procedure

The survey was available on the Internet at from the 25th of November 2017 until the 4th of December 2017, as a topic appropriate for this website’s readers, given the approaching “end” of 1 year and the beginning of a “new” one. The survey was in Norwegian and opened with the following question as a catchy title: “Friends: Help! If the year is a circle – where’s March and December in your mind?”^1^. Participants viewed an orange empty circle on screen (about 10 cm in size on a standard screen) and placed a “month” label by “dragging” a dot to the “correct” location on this abstract circle. This was performed for the month of December, and then for the month of March, so that these positions provided a continuous and direct measure of the imagined position of these months on a circular spatial model.

Respondents could then continue, if they wished, to fill out a questionnaire composed of drop-down windows or multiple choices that they could select with a mouse (or finger with mobile phones). In the first item of the questionnaire, respondents could actually select one of the 12 clock positions (in text form) for the month of December or select “I cannot answer this.” In the second item, respondents followed the same procedure for positioning the month of March. These first two items provided a “categorical” set of spatial locations closely related to the analogy of the clock face. These choices may also clarify what type of temporal “anchors” an individual may have used in the previous dot positioning task. In fact, when asked where December is, we did left unspecified which particular “point” within this month one should use as “anchor” and different people may have decided to use the beginning, midpoint, or end of December as time anchors. The initial questions related to the overarching question of “What does your inner image of a year look like?” Answers were either multiple-choice selection, or typed as free text. Based on the first anecdotal findings by the colleague who posted a piece of paper with a circle, we expected that many participants would be able to imagine the year as a circular shape. At the beginning of the test, all respondents could indicate, by dragging a month label on the circumference of a circle, where the positions of December and March would fall within their own year’s imaginary circle. They were later asked to translate the spatial data to 12 possible positions on a clock face (e.g., March at about “03:00” o’clock). Some participants also provided drawings or graphical representations of their preferred mental image. Respondents’ typed free text responses about the preferred shape of their “inner year model” that were tabulated subsequently in six main shape classes: “circle,” “ellipse,” “spiral,” “line,” “square,” or “other.” Crucially, we collected each individual’s rating of the degree of “naturalness” in imagining the year as a circle. Respondents were grouped based on their self-declared demographic characteristics for age, gender, handedness, geographical location (e.g., region of origin in Norway), and the role of these factors were examined in statistical analyses.

In the subsequent three items, we probed how “natural” it felt to each respondent to think of the year as a circle (by clicking on one of a series of 5 numbered circles: 1 = “very unnatural”; 5 = “very natural”). A similar 5-step Likert scale was used to probe whether it was actually possible for the respondents to imagine a different spatial system, where December would be in a different position and the year “move” in the opposite direction (1= “it is completely impossible”; 5 = “it is super simple”). The next item directly posed the question “Do you imagine the year as an even circle, or does it have a different shape?” In this case, either the respondent could select “an even circle” or “other” as responses and in the latter case freely type in the name of their preferred shape. Then the item below asked respondents to type freely a response to the question “Do you have any theories on why you perceive the year in this way?” In the following item, we collected information on whether respondents had ever thought about the question of a spatial image of time, by selecting three possible answers (“I’ve been thinking about it, and have spoken with others about it,” “I’ve been thinking about it for myself earlier,” “I’ve never thought about it before”). Next, we questioned whether the respondent had previously encountered so-called “Year planning wheels” at work, school, etc., by selecting between three choices which specified the direction of rotation of the wheel (“Yes, they tended to rotate the same way as mine,” “Yes, they tended to rotate the opposite way of mine,” “No”). The rest of the questionnaires asked participants to indicate, by selecting among multiple choices, their handedness (“right-handed,” “left-handed,” and “other”), gender (“female,” “male,” “other”), age group (0–11, 12–15, 16–19, 20–29, 30–39, 40–49, 50–59, 60–69, 70–79, 80+). A few additional items requested the respondents to rate themselves according to a few colloquial personality traits (e.g., creative). The final item of the demographic information, was answering the question “In which part of the country did you grow up?”, selecting between five regions, or entering free text. At this point, respondents could submit the whole set of answers (whether they had completed the whole questionnaire or left blank several items) by pressing the “send” button at the bottom. The data set is publicly shared at https://nrkbeta.no/2018/01/30/lagnoefint/ (by clicking on “*ÅrFinalPublic*”). Nota Bene: Although we could tabulate most of the responses directly as they were, since they required selecting one out of multiple choices, we classified retrospectively several other responses based on the typed text. In particular, several participants who chose shapes “other” than circle as their preferred way to imagine the year wrote longer descriptions, which we attempted to classify into other generic groups. For instance, we considered each instance in which our Norwegian respondents used the word “firkant” (i.e., four-edged) as indicating both square and rectangular shapes, as it happens in colloquial Norwegian language. Similarly, “square” in the following analysis indicated any rectangular shape. However, we distinguish from the “even circle” other circular shapes that were particularly prevalent like the ellipse (combining ellipse, oval, flattish circle, etc.) and the spiral.

## Results

The data consisted mainly in frequency “counts” based on the respondent’s selections to each of the multiple-choice questions. Hence, we analyzed results mainly with non-parametric tests that compared the observed with the expected frequencies; i.e., *chi-squared* (*χ*^2^) tests or *binomial* distribution tests using either the *JASP* software^2^ or *Statview*. For ratings (e.g., “ease of imagining the year as a circle”), we used standard ANOVA with the average scores as dependent variables. Given the very large sample, we expect that most statistical comparisons will turn out to be (extremely) significant despite their little substantive or even trivial effect sizes (Cohen, 1990; Sullivan and Feinn, 2012; Lantz, 2013). Hence, for the *chi-squared* (*χ*^2^) tests, we computed the Cohen’s *W* (i.e., the square root of the *χ*^2^ value divided by *N*) and the *post-hoc* Pearson’s cell contributions (cell’s *p*), standardized residuals that indicate what each cell in the observed frequencies contributes to the chi-square statistics. For Cohen’s *W*, a value of 0.1 is a “small” effect size, 0.3 is “medium,” and 0.5 a “large” effect size. For the cell’s *p*, a value greater than 1.96 (for *p* < 0.05 significance) indicates that the cell provides significant information about occurrences being different from what one would expect under the hypothesis of independence. For ANOVAs, we provide the effect size, either Cohen’s *d* or *partial eta squared* ($\eta_{p}^{2}$).

### Placing the Dots

**Figure 1:**
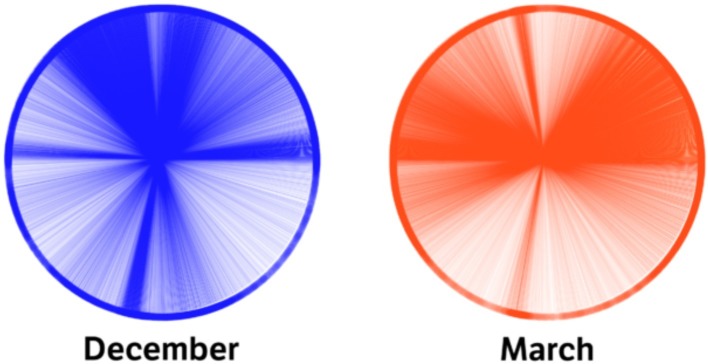
Wheel diagram of 76,922 placements of the months of December (left blue diagram) and March (right red diagram) on the circumference of an empty circle. Graphics realized by Henrik Lied at NRKbeta.

Responses to the first item consisted of pixel-grained positioning, with the mouse or finger, of a month label on a circle presented on screen. Thus, we first simply visualized these responses by plotting them on a “wheel diagram” (see Figure 1) where each of the spokes or radiuses represents one selection by a particular individual (Nota Bene: the heavily colored sections of the circle represent time regions where a large number of respondents selected the same or adjacent angular positions).

**Figure 2:**
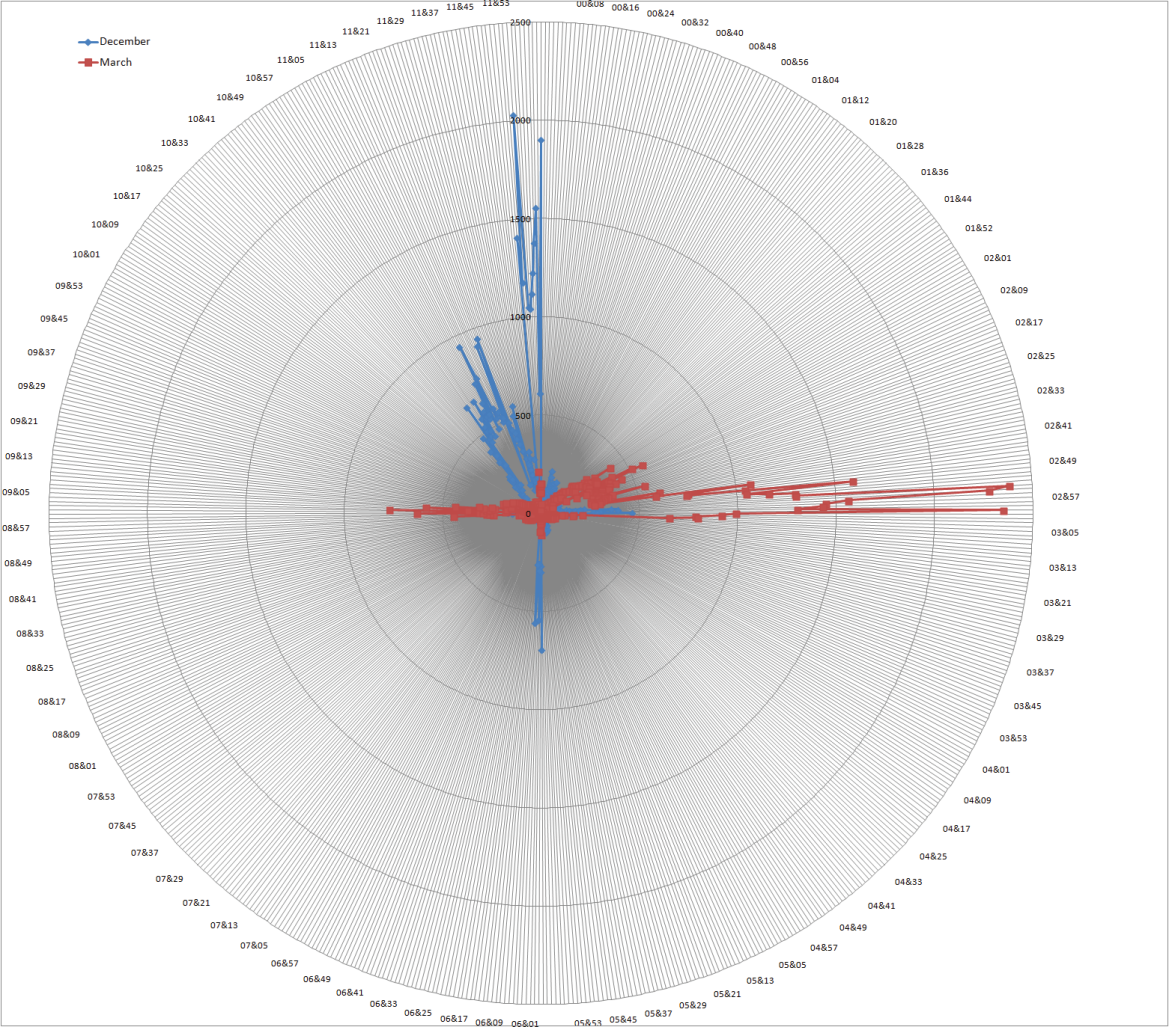
Wheel diagram of 76,922 placements of the months of December (blue graph) and March (red graph) on the circumference of an empty circle. The digits around the circle correspond to the closest minutes’ positions on a clock face and the concentric circles represent frequency rates of selection for a position in steps of 500 clicks.

The wheel diagram in Figure 1 clearly reveals that there was a remarkable variability in the positioning of the 2 months, with nearly every possible location on the circumference chosen at least once. This in itself is evidence that not all respondents do the spatializing task according to some standard blueprint, available in the culture, but that there is room for idiosyncratic expressions. However, as shown in the next wheel diagram of Figure 2, of the 76,922 placements of the months of December (blue graph) and March (red graph) on the circumference of an empty circle, there were clear remarkable differences in the frequency of specific locations and systematically for each of the months. As displayed in the clock- labeled image of Figure 2, segments of the circumference tended to be selected at very high rates (e.g., between 500 and 2,000 individual choosing the same position), with clearly delineated subgroups of individuals crowding their selections close to some specific position within each segment of time.

Most remarkably, Figure 2 reveals several peaks in the frequency of locations: For December, there are two peaks around the 11:00 and 11:30 positions and a large peak around 12:00 o’clock. A similar asymmetry in positioning is also present, though less saliently, for the month of March: with a smaller peak around the 2:00 position “anticipating” the larger peak at 3:00 o’clock. Hence, there may be a general tendency to view the ending of the month as the salient landmark (cf. Teigen et al., 2017). Importantly, Figure 2 reveals two peaks in the opposite directions from the canonical positions of 12:00 and 3:00 o’clock for December and March, respectively. These 6:00 and 9:00 o’clock peaks (when collapsing choices within ± 15 min) were smaller (*N* = 4,150 and 7,338) than the 12:00 and 3:00 o’clock positions (*N* = 16,178 and 27,978), representing about a quarter of the choices for the canonical positions.

### Responses to the Questionnaire

A good portion of the respondents (71.4%; *N* = 54,927) to the above dot-positioning task also chose to continue responding to our questionnaire. First, we deleted empty posts and responses that were clearly jocular. We also deleted identical replies posted within a short timeframe so that of identical responses, entered several times in a row, we included only one reply. The final data set on which we base the following analyses consisted of 39,797 posts. Among these individuals who continued to fill out the online questionnaire, the great majority (*N* = 39,423) specified their gender either as “women” (*N* = 21,472) or “men” (*N* = 17,951). According to a binomial test, the proportion of females (54.5%) was significantly greater than that of males (45.5%), *p* < 0.001. As expected, among the respondents that indicated a specific handedness (*N* = 39,617) about 90% of the respondents (*N* = 34,784) declared to be right-handed (left-handers: *N* = 3,860 or 10%). People in their 30’s constituted the largest group of respondents in terms of age (*N* = 11,291) followed by people in the 20’s (*N* = 9,135) and 40’s (*N* = 9,103) and, in descending order, the ones in their 50’s (*N* = 5,431), 60’s (*N* = 2,664), under-20 (*N* = 1,110), and over-70 (*N* = 883).

### The Shape of the Year

**Figure 3:**
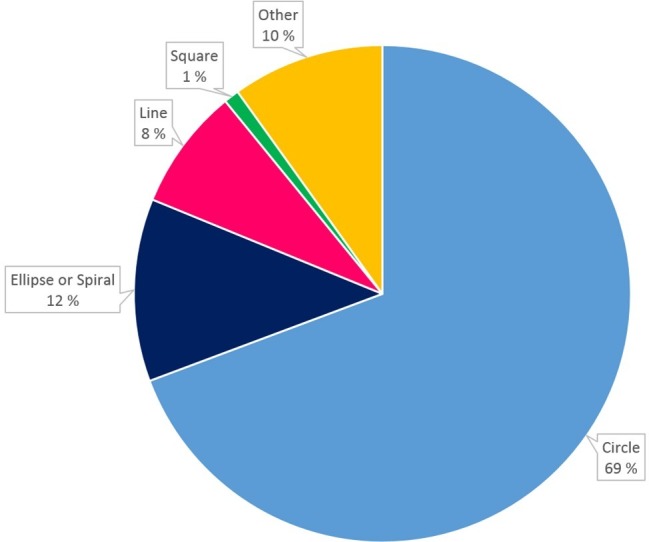
Pie chart of proportions of individuals who indicated a particular shape corresponding to the mental image of the year.

Among the respondents that specifically named a particular shape when answering the item “Do you imagine the year as an even circle or does it have a different shape?” (about 2% left this item blank), the great majority (*N* = 27,814 people or nearly 70%) indicated the default “an even circle” as their imagined shape (see Figure 3), whereas all other shapes (ellipse or spiral: *N* = 4,851; line: *N* = 3,140; square: *N* = 394; or other: *N* = 3,867) were chosen in much smaller proportions (1–12%).

A binomial test confirmed that only “circle” had a proportion of choices greater than 0.5 (*p* < 0.0001). Interestingly, about 10% of the participants indicated spatial shapes that were either not easily classified in the typical shape categories like “circle” or “line.” These were lumped together as “other” in the analysis. These could include simple graphical motifs like figure-of-8s, waves, rubber bands, U shapes, etc., as well as longer, free-text mini-essays about complex, highly personal narrative visualizations, for instance with the months arranged around the garden outside the house where the respondent grew up.

### Naturalness of a Circular Temporal Form

**Figure 4:**
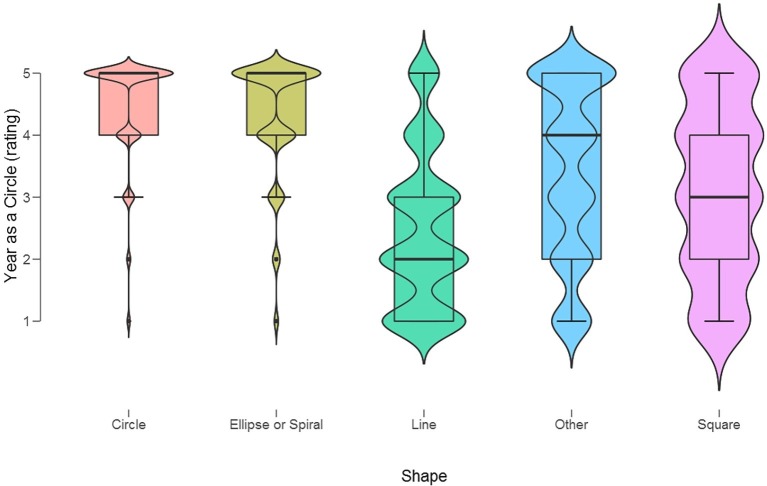
Violin boxplots of the naturalness of “seeing” the year as a “circle” in individuals who had chosen different shapes (x-axis) as their preferred spatial models.

Respondents also indicated how “natural” or easy was to think of the year as a circle by using a 5-step rating scale (1 = very unnatural; 5 = very natural). We performed an ANOVA with Shape (“circle,” “ellipse or spiral,” “line,” “other,” “square”) as the independent variable, indicating which shape was actually preferred as spatial model, and Years as a Circle (rating) as the dependent variable. This revealed a significant difference, *F*(4) = 3,322, *p* < 0.0001, $\eta_{p}^{2}$ = 0.25, or a “very high” naturalness of the “circle” model (see Figure 4) for individuals who had originally chosen either the “circle” (Mean = 4.47, SD = 0.9) or “ellipse or spiral” (Mean = 4.30, SD = 1.0) as their preferred spatial models. In contrast, the “circle” was rated “unnatural” by those who had chosen the “mental line” model (Mean = 2.5, SD = 1.2). Interestingly, respondents who had chosen the “square” model (Mean = 3.1, SD = 1.4) or “other” shapes (Mean = 3.4, SD = 1.5) did not, on average, find it either unnatural or natural to imagine the year as a circle. Indeed, when probing how possible it would be to imagine the months in a different spatial system with an opposite direction of movement of the year, individuals who chose “circle” were close to neutrality regarding imagining an alternative year shape, since their mean ratings were 2.64, SD = 1.34, on the 5-step Likert scale (1 = “completely impossible,” 5 = “super simple”).

### Gender and Circular Time

A *chi-squared* test was performed on number of respondents split by Gender (female, male) naming a particular shape (“circle,” “ellipse or spiral,” “line,” “other” “square”). This revealed a significantly different distribution of preferred spatial models across genders, *χ*^2^ = 83.5, df = 4, *p* < 0.0001. The *post-hoc* Pearson’s cell contributions confirmed a significant increase in females’ spatial models of curvilinear shapes like “ellipse or spiral” (cell’s *p* = +7.3) than the standard circle (cell’s *p* = −4.0) coupled with a decrement in favor of the “mental line” in this same group (cell’s *p* = −4.7). The opposite pattern was seen with males, since relatively fewer chose other curvilinear shapes (cell’s *p* = −7.3) and more subscribed to a “mental line” (cell’s *p* = +4.7). However, this gender-related effect had very small size (based on a Cohen’s *W* = 0.05).

### The Planning Wheel

To assess the influence that having seen before, at work or school, a “year planning wheel,” we performed a *chi-squared* test on number of respondents who answered affirmatively or negatively to using a planning wheel, and their respective naming a particular Shape (“circle,” “ellipse or spiral,” “line” “other, “square”), *χ*^2^ = 615.5, df = 3, *p* < 0.0001. Indeed, having seen before a concrete example of spatialization of the year as a “wheel” increased the probability of naming the “circle” as the preferred spatial image (*N* = 13,837; cell’s *p* = +20.1), compared to respondents without such experience (*N* = 13,125). Moreover, it decreased the frequencies of naming any of the other shapes (“ellipse or spiral”: *p* = −4.1; “line”; *p* = −23.5; “square”: *p* = −2.3). The Cohen’s *W* = 0.15, indicating the presence of a moderate (small to medium) effect of previously using a planning wheel. In addition, 52.7% of respondents (*N* = 18,846) had never thought about the year, at least explicitly, as a circle whereas 47.3% did. Interestingly, having imagined or not in the past the year as “circular” had no effect on such “distance,” *F*(1,27,598) = 6.47, $\eta_{p}^{2}$ < 0.0001.

### Categorical Clock’s Positions

**Figure 5:**
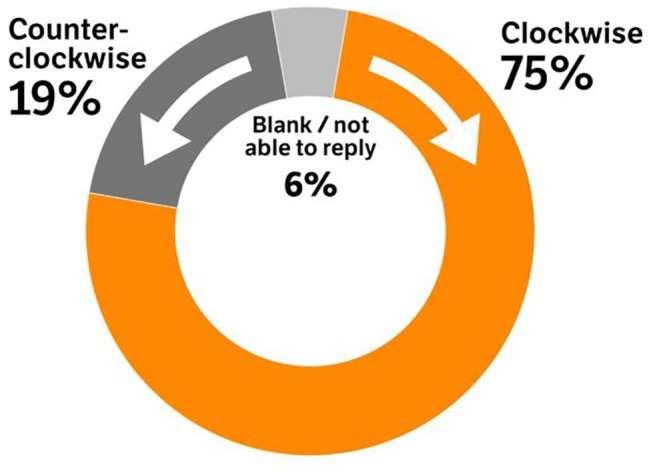
Proportion of respondents choosing opposite direction of time on the year’s wheel. Graphics by Vidar Kvien, NRK.

Interestingly, the relative, “categorical,” “clock” positions of December and March allowed reconstructing *post-hoc* the time’s direction of motion within the spatial model. As illustrated in Figure 5, this computation revealed a strong preference for the clockwise direction (*N* = 29,881) over the counter clockwise direction (*N* = 7,758), binomial test: *z* = 114, *p* < 0.0001. We expected the presence of a handedness bias, perhaps based on the preferred direction of manual torque of the dominant hand, which could engender a general preference for an imagined direction of motion. Hence, we performed a *chi-squared* test with handedness (left-handed, right-handed) and Rotation (“clockwise,” “counter clockwise”). This revealed a significant difference among groups, *χ*^2^ = 22.4, df = 3, *p* < 0.0001. Contrary to expectations, among individuals that arranged the year’s months in a counter clockwise fashion on the circle, there were relatively fewer lefthanders (*N* = 687) than the expected frequency (*N* = 755), or relatively more righthanders (*N* = 6,877) than expected (*N* = 6,808). However, the Cohen’s *W* = 0.054, indicated a very small handedness-related effect.

The first item of the questionnaire allowed respondents to fit explicitly the locations of the months of December and March in the “scaffold” of the 12 h categories. A group of respondents chose 11:00 for the December position (*N* = 14,417), possibly marking in this way the end of the December (or New Year) with the top, cardinal, 12 o’clock position. The latter clock position was chosen by a slightly smaller group (*N* = 13,053). Most respondents positioned March at 3:00 o’clock (*N* = 19,018), but there was also a slight tendency by a smaller group of respondents to “anticipate” it 1 h earlier at 2:00 (*N* = 7,719). The anticipation may either reflect the same process of giving saliency to the months’ endings or simply using the months’ numbering in the calendar, also ensuing a constant distance of 3 steps between the 2 months.

Indeed, there was overall a tendency to locate the months as three “hours” apart from one another (*N* = 24,716 or 62% of cases). However, there was also a great deal of variability within the population of respondents, which may be exposing individual differences in the subjective sense of duration or lapse between the salient temporal landmarks characterizing these 2 months. When we used these “hour” positions to compute an estimate of the temporal “distance” (in “hours”) between the 2 months, for a subgroup of participants the distance between December and March corresponded to 4 “hours” (*N* = 10,124 or 25%) or 2 “hours” (*N* = 2,068 or 5%). In fact, based on the average lapse for the whole sample, the distance between December and March tended to slightly exceed equidistance (Mean “D-M distance” = 3.24 h; SD = 0.66).

### Age and Duration

We checked whether the above estimates of subjective duration were related to the age of the respondents. We found that those “above 60” had a slightly shorter “distance” between the positions of December and March (Mean distance on the clock face = 3.18 h, SD = 0.71) than those “under 60” years of age (Mean distance on the clock face = 3.24 h, SD = 0.66). The size of this effect was however small (based on a Cohen’s *d* = 0.09) and therefore negligible. In order to test the prediction that older individuals would be more likely to image the year as a “circle” and less as a “mental line,” we collapsed age groups into two categories: Individuals “above 60” and “under 60” years of age. We then compared with a *chi-squared* test, the proportion of choices in these two age groups for either the “circle” or the “line” (“above 60”: circle = 2,725; line = 160; “under 60”: circle = 25,033; line = 2,954). There was a significant decrease in the selection of the “line” as spatial model in individuals “above 60” (cell’s *p* = −8.5) coupled with an increased selection of the “circle” in this same group (cell’s *p* = +8.5), *χ*^2^ = 71.8, *p* < 0.0001. However, the Cohen’s *W* = 0.05, indicates a small effect size of old age. A linear regression between each participant’s age (in years) and “distance” showed no relationship, *R* = 0.03.

### Geographical Location

Finally, in a *chi-squared* test, we compared the proportion of choices for shapes (circle, ellipse or spiral, line, and square) depending on whether the respondents grew up in either Southern (*N* = 27,037), Central (*N* = 3,761), or Northern Norway (*N* = 3,776). Although there was a significant effect, *χ*^2^ = 35.8, *p* < 0.0001, showing variation across locations, there was only a very small increase in the observed frequency of “circle” (*N* = 2,991) over the expected frequency (*N* = 2,928) by people who grew up in Northern Norway. Again, the Cohen’s *W* = 0.032, indicates little support for an effect of geography.

## Discussion

This may be the first study to probe representations of time in an understudied European population that shares one language (Norwegian) despite its nation’s large geographical extension in latitude, with some of the northernmost urbanized locations in the world. Given that 152,000 website visitors saw the initial request on the website, a response rate of 76,922 placements of the months on a circular display implies that about one out of two readers of the website chose to participate voluntarily to the present survey. This astonishing high rate leads us to consider that a large portion of the population thought that the survey addressed something important or relevant – something about lived time and their minds. Clearly, the suggestion that the year could be a “circle” was meaningful to many readers.

**Figure 6:**
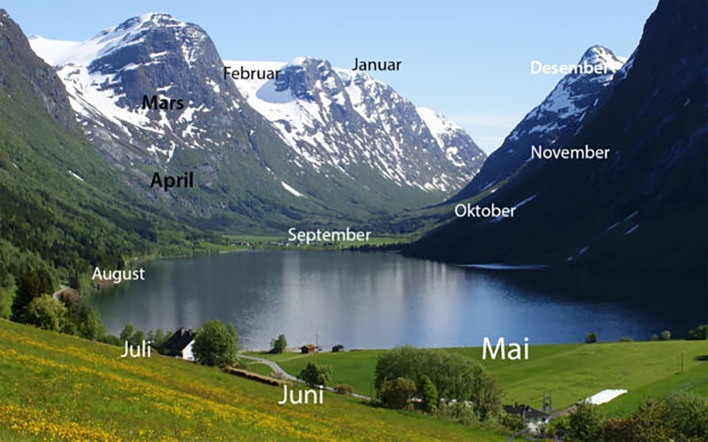
Image sent by one of our respondents, indicating a highly idiosyncratic spatial organization of the year’s months (in Norwegian) superimposed onto a familiar landscape. Illustration by Ola Hansen/Algkalv CC BY 3.0.

Based on (1) the historical expressions of the concept of the year in diverse human, material, cultures, and (2) the evidence of previous studies, like Leone et al.’s (2018), one could expect the present survey to reveal a general propensity or ease in imagining the year’s units of time (e.g., months) as arranged within imaginary “wheels.” Indeed, it was clear from the results that, at least within Norway (a Western, economically affluent, literate society), it may be natural if not common (i.e., 70% of 39,797) to imagine the passing of a year as occurring along a curved path, closed on itself like a circle, and to visualize such a curvilinear time as moving clockwise (i.e., 75%). Based on our responses to a questionnaire, where participants rated the naturalness of a circular spatial frame, most of our respondents did not find it strange at all to position months into a circular shape as shown in Figure 1. Hence, the present results provide evidence that spatializing certain aspects of time does not occur exclusively in terms of a mental line within a literate, Western, society like Norway. There was nevertheless variety in our sample with regards to the mental images attributed to the year and about one third of the respondents preferred other shapes than the simple “circle,” though some were still curvilinear (like ellipses or spirals) but others clearly linear or rectangular, occasionally resembling polygons. About 10% of the participants indicated spatial organizations that were very idiosyncratic, linked to particular images or even to the landscape (as indicated by the image in Figure 6, posted by one of our respondents). These findings reinforce that idea, put forward by many scholars from a variety of academic fields (archeology, social anthropology, linguistics, semiotics, and psychology) that humans think about time in terms of spatial images. These “images” can literally take the form of “visual images,” which are clearly represented in space, or at least in the form of a “mental model.” Given the radical difference between our so-called sense of time and our actual sensory information (e.g., visual and tactual), a radical account of the sense of spacetime is that our mind have no other cognitive tool at disposal for organizing the experience of succession of events than a spatial framework of some kind (e.g., elapsed time by distance in space).

However, people may differ widely regarding which particular spatial frame they may find most appropriate for the momentary task of thinking, reasoning and talking about time. In the present study, we focused on a long-range concept of time, that of the year, which although it is to some extent an arbitrary unit of time, it has clear foundation in cyclical natural phenomena. We consider those changes in our perception of astronomical bodies (the Sun, Moon, and star constellations) as well as a plethora of changes in the physical environment (the seasonal changes in daylight, vegetation, food supplies, animal behavior, temperature, etc.), all these may contribute to “shape” our images of spacetime. Different cultures contribute with additional social factors, temporally anchored to some of the above environmental factors, and therefore “cyclical” social events (e.g., New Year’s Eve, Midsummer Night, etc.). Indeed, due to the cyclical aspect of these long-term temporal changes, many of us should find a wheel-like, curvilinear, spatial form to be most representative or practical for thinking and displaying the passing of time during a year.

We would suggest that the present evidence indicates that representations of spacetime could flexibly adapt, according to several characteristics of our respondents and task demands. For example, given previously available spatial models (e.g., the “year planning wheel,” the wall calendar, the wall clock), as well as the specific direction of reading in the native language. Previous studies on temporal relations like past, present, and future have revealed that it seems natural to use a “linear” topology for these aspects of lived time, so that this appear to “flow” along a horizontal line from left to right (i.e., consistent with direction of reading in European languages). Occasionally this line has an upward diagonal slant (i.e., inconsistent with reading in European languages), as measured by positioning labeled shapes (Leone et al., 2018) or by monitoring eye movements (Hartmann et al., 2014). Interestingly, the modern and “ubiquitous” wall calendars preserve in a square-like matrix the unfolding of time in linear rows from left toward the right (Monday–Sunday) and from top to bottom for weeks. Time may seem to flow in Western cultures from an egocentric viewpoint (e.g., left or right with reference to the speaker; Núñez and Cooperrider, 2013); however, a few societies use an allocentric spatialization based on environmental features (e.g., east or west; uphill or downhill) regardless of the direction faced by speakers (e.g., McGlone and Harding, 1998; Fedden and Boroditsky, 2012; Boroditsky and Gaby, 2013*).*

One can also apply a rectilinear topology to the spatial representation of months (e.g., Gevers et al., 2004). Nevertheless, as testified by the present results, “non-linear” and “circular” topologies would seem natural for iterative or cyclical temporal relations as well, like for seasons or parts of days (Leone et al., 2018), weekdays and months (Seymour, 1980; Zorzi et al., 2006). As shown in the present survey, a large proportion of respondents confirmed to subscribe to a circular spatial image for the year. Even those who explicitly stated their preference for non-circular spatial forms “seemed comfortable” (based on their ratings of naturalness) using a circular topology. Interestingly, shapes like the ellipse or spiral also had a higher representation among those who had reflected on the year earlier, indicating that deliberate reflections on the shape of the year may lead to spatializations that are more detailed and personal. A few respondents indicated in the free text field that the spiral form allows the year to be repetitively cyclical but also conserve a linear progress, providing an apt “hybrid” spatialization that reconciles the rhythmic feature of the passing of years with the linear “arrow of time” and that may reflect subjectively the different durations of the seasons.

We note that directionality biases in action and path of motion are documented for both animals and humans (Karim et al., 2016). Organisms have a tendency to turn when walking or rotate parts of the body in a specific direction (e.g., turning the head to the right, Güntürkün, 2003) and some of this right-turning tendencies may be stronger in righthanders than lefthanders (Scharine and McBeath, 2002). In the Norwegian language, the direction of reading is the standard, Western, left-to-right, downward, motion pattern. Interestingly, right-handed respondents assigned higher naturalness to the “circle” model than lefthanders. As pointed out by Lugli et al. (2018), the clockwise and counterclockwise circular movements might represent a strengthened horizontal movement to the right and to the left, respectively. We had considered that manual “torque” (or the force exerted when acting on many objects that require a rotational movement of the hand or wrist or arm) could have led righthanders to imagine the cyclical motion of time in a clockwise direction; while lefthanders might prefer the opposite motion. Instead, we discovered a weak but reversed trend, since there were more righthanders that reported a counter clockwise preference or more lefthanders reporting a clockwise preference than expected. These findings should however be considered cautiously since the size of such a handedness-related effect was very small. We also found little support for an influence of geography (due to experiencing a complete circular sun path in the summer at Northern latitudes; Laeng et al., 2007) on either shape or direction of temporal movement.

Interestingly, we observed a slightly higher preference for the “ellipse” or “spiral” in women, although we should also keep in mind that the overall gender-related effects were very small. A cyclical image of time may come more natural to women, but another possible explanation is a gender-specific openness to discussing personal experiences. In general, female respondents had a higher representation among those who had thought about the shape of the year and discussed it with others. We have more female respondents than males in this survey but, interestingly, there is no overrepresentation of female internet users in Norway, NRK.no, or *NRKbeta*. The gender bias may simply reflect a keener interest for the topic or phenomenon in women than in men.

Respondents found it natural to map the months on a clock-like representation and there was high consensus in numbering the months in the same way as the hours on a clock, from 1 to 12 (March = 3 and December = 12). Indeed, when computing the distance in “hours,” the majority of respondents (64%) attributed a 3 h distance between 2 months. However, the influence of the clock as cultural artifact was not absolute since the data also reveal different “distances” to the passage of time between December and March. On average, there was a slight tendency in the population to overestimate the passage of time between December and March. Selections were skewed toward positions that either preceded or coincided with the month’s cardinal number, but comparatively few selections are seen after this point (e.g., between 12:00 and 1:00). It is possible that respondents imagined attaching an imaginary word label (e.g., a sort of mental “post-it” tab) to a vertically oriented circle, hence positioning the midpoint of the month to so that the extent of the month would best overlap onto the corresponding segment of the “year” circumference. If so, an asymmetry toward the “left” of the cardinal 12 position (i.e., prior in time) may also indicate that for this month, the relevant categorical border is at the “end” and not at the “start” or midpoint of the month (or year), that is at the transition to New Year. Since a similar asymmetry, albeit weaker, was also present for the month of March, we surmise that there may be a general tendency to view the border between the ending of a particular month and the beginning of the next (i.e., the transition) as the salient landmark. In a study on mental representations of time events, beginnings appear to “loom larger” than endings, since they attract more attention and seem more important and interesting than the end of the same event (Teigen et al., 2017). In the present case, one may consider December as “the” central (and top) month in our calendar because it marks the end of the year but also the transfer to a new year and a major beginning. Moreover, December looks ripe from a “socio-time” view, since it also contains the salient Christmas period (“Jul” in Norway) near its end and the New Year’s beginning. This in itself may give to December prominence over other months, which may influence its imagined size and expanse within a spatial ring model (Seymour, 1980; Leone et al., 2018).

**Figure 7:**
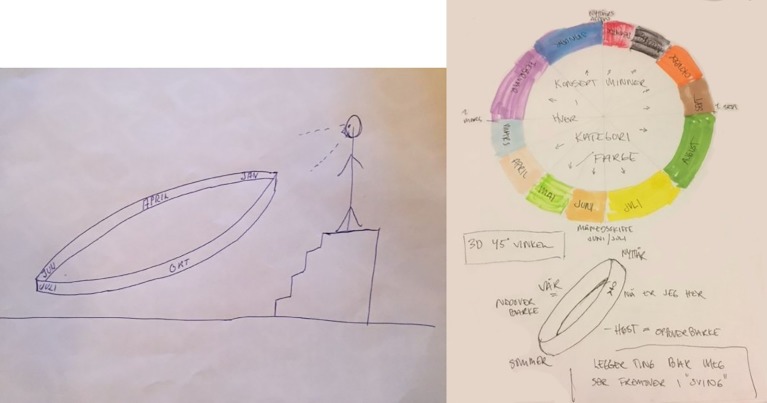
Drawings submitted by survey respondents illustrating their “calendar synesthesia” where the year is a ring or wheel oriented at a specified angle and elevation in relation to the synesthete (left panel) and for one who reports specific colors for each segment of the “year ring.”

As Figure 7 illustrates, in two graphic examples provided by two different respondents, a person may visualize the cycle of months as a ring in extrapersonal space (see left panel) or as a path where one is located within one of its segments corresponding to a month (see right panel). Interestingly, one may attribute different size to each segment so that the distance between different months’ borders may vary (see right panel). Such variability between months’ length might explain why December’s positions gravitated over the 11 and 12 o’clock positions, whereas March may have held a more stable anchoring at 3 o’clock (also corresponding in number to the third month in the calendar). Thus, objectively identical periods may be different in perception due the saliency of events occurring within the same temporal window (Klein, 2006). It is also interesting to note that research on the subjective perception of time duration have suggested that: “life speeds up as we grow older” (Block et al., 1998; Wittman and Lehnhoff, 2005; Arstila and Lloyd, 2014; Wittman, 2016). Although the existence of this effect remains controversial and it may just be a temporal illusion or “false belief” (Wearden, 2015), it is worth considering it in the present context, since it could play some role in yielding the slight reduction of the December–March “distance” in the mental images of respondents above 60 of age. Interestingly, having imagined the year as a circle previously did not have an effect on the “distance” measure.

One may suggest that the present study might indicate some form of universal “synesthesia,” since a great majority of respondents found it natural to think of yearly time as a circle. It is likely that a subgroup of respondents in this survey did include individuals who show a form of synesthesia dubbed “calendar synesthesia” (about 2.2% of the general population, by an estimate of Brang et al., 2010). However, our sample was self-selected and therefore it is possible that there was an over-representation of synesthetes. Researchers also debate whether synesthesia is a trait that varies continuously in the human population, so that the “synesthetes” are individuals at one extreme end of the distribution of cross-modal associative abilities (Martino and Marks, 2001; Marks, 2011; Deroy and Spence, 2013; Cohen, 2017; Deroy, 2017). Alternatively, these similarities are superficial and synesthetes consist of a separate, categorically different group, reflecting a particular neural condition (and genetic profile). However, there seems to be a salient qualitative difference between (1) anyone’s ability in generating a mental image of days or months in a circle and (2) the synesthete’s report of “seeing” them (e.g., Sagiv et al., 2006; Tammet, 2007; Hubbard et al., 2009; Simner, 2009; Brang et al., 2010; Jonas and Price, 2014).

For a “calendar synesthete,” days or months can be located in specific places and distances to the body and as objects or regions with unique colors (Smilek et al., 2007; see Figures 6, 7 by our respondents). In the latter case, the synesthetic image has an embodied “presence” that can have considerable consequences on the synesthete’s richness and vividness of memory for specific episodes (e.g., Luria, 1968) as well as on the cognitive manipulation of time-based information (Mann et al., 2009). Moreover, a study of 571 cases of self-reported “spatial sequence synesthesia” (Eagleman, 2009) suggests that the most common temporal shape for synesthetes is a line (27%) and that the preference for circular arrangements of months may be lower in these special individuals (fewer than 20%) than it may be in the general population.

One of the most interesting findings in the present survey is that, when respondents were explicitly queried in the questionnaire section about their imagined geometry of “year,” a rather small minority of individuals (about 10% in the present survey) mentioned the so-called “mental line” (i.e., a mental representation of succession; e.g., Bonato et al., 2012) as the preferred spatial framework. We cannot exclude that such a low percentage may simply reflect the fact that we prompted or “primed” all respondents from the start to think according to a circular framework. In fact, a popular method for revealing how one thinks about time is to provide spatial primes (e.g., Boroditsky, 2000). Yet, it is just as possible that most individuals will reveal a preference for circular spatial models when thinking about the year as a cycle, regardless of the format of the questioning (as indicated by the findings of Leone et al.’s, 2018).

Intuitively, linear versus non-linear time are contradictory and cannot be used to express the same thought at the same time. Hence, one may opt for either one form or the other, depending on whether the focus is on time as a cycle of repeated events or a series of unrepeatable events (i.e., a lost past and an utterly unknown future). It may not be accidental that when speaking or gesturing, so many individuals (Núñez and Cooperrider, 2013) refer to the past as behind us or below us (i.e., gone) and the future ahead of us or, if one lives in a valley, uphill (i.e., approaching). However, such a relationship between ahead/behind and past/future is reversible (e.g., Núñez and Sweetser, 2006) and, if the focus is on “visibility,” the past can be reminisced while the future is not yet visible. Hence, we surmise that most of us are already used to switching from one spatial form to another if the context requires so (e.g., Torralbo et al., 2006; Casasanto and Bottini, 2013; Flumini and Santiago, 2013; Duffy, 2014). Indeed, we observed that many individuals who had indicated the “line” as their preferred spacetime form could nevertheless position, with self-declared “moderate ease,” the temporal relations of a year onto a clock face-like display. Moreover, the long timescale of a year might make the “shape” of the entire year feel imperceptible from an everyday perspective, in the same way one does not sense the curvature of the earth while walking.

A flexibility of mental models might possibly explain the seeming dominance of the circle model in our society. Analogously, in language, individuals can take different perspectives when talking about spatial relations, as well as spacetime relations, this excluding viewing cultural preferred spatial frames/forms as “strongly” shaping concepts of time as in the Whorfian relativistic perspective (Gell, 1992; Pinker, 2007). We also saw that having seen before a concrete example of spatialization of the year as a “planning wheel” increased the probability of naming the “circle” as the preferred spatial image, which suggest that people’s perspectives on the movements of events in time are also influenced in their interactions with specific cultural artifacts (Duffy, 2014). Several non-western cultural systems (Hendry, 2003; Loewe, 2003) seem to support a conceptualization of temporal progression as a cyclical process (e.g., in Hinduism or Buddhism), where unescapable key events repeat themselves in the “eternal return” of mythical time. These can also be externalized in artistic drawings (or sculptures) of “wheels” or “rings” of time (e.g., as the Hindu *Saṃsāra* “wheel of existence”, the Ouroboros, the Zodiac, the Babylonian or Mesoamerican sun wheels, the “wheel of the year” of Neopaganism; see Lima, 2017). These cyclical periods are evidenced from practices related to the “reproduction” of the societies, as embodied in farming and ritual practices (Lucas, 2005, p. 68). A conceptualization of time in cycles goes well beyond creation myths and farming rituals however, since even modern physics has entertained the idea of an endless series of *big bang*s followed by *big crunch*es (e.g., Steinhardt and Turok, 2001*)*. Several natural phenomena are iterative, like the decay and renewal of plants (which conflates irreversibility and its opposite), the migrations of animals, the freezing and melting of ice, all leading toward an iterative spatial model where time curves on itself. The “long range” biorhythms of the living world (e.g., the seasonal changes of reproduction and birth in animals, their migratory cycles, the female menstrual cycle), as well as the human “life cycle” of generations repeating the same developmental milestones (Yamada and Kato, 2006), all of these represent strong, external, causal events underlying the representation of time in all cultures (Gell, 1992).

One conclusion of the present study is that the relationship of spatial cognition to time cognition appears to be manifold and flexible not only across cultures but within a same culture (Bender and Beller, 2014). Previous studies had shown that the capacity to learn a variety of mappings between spatial forms and temporal sequences is present in most individuals in western culture (Brang et al., 2010). Moreover, current cognitive neuroscience has shown that the brain’s representation of space uses multiple, parallel, forms of frameworks (e.g., egocentric, allocentric, object-centered, environmentally centered; e.g., Laeng et al., 2002). At least two fundamental types of neural codes have been identified: a “coordinate” (or “quantitative,” “analogical”) as well as a “categorical” (or “qualitative,” “digital”; e.g., Kosslyn, 1994; Laeng and Peters, 1995; Laeng et al., 2003; Laeng, 2013). Areas of the brain processing spatial information (e.g., the parietal regions) may also be keys to judgments of “when” things happen, beside “where” (Harrington et al., 1998; Battelli et al., 2007; Gauthier and van Wassenhove, 2016).

We surmise that the fundamental dichotomy between analogical-digital representations of space (Kosslyn, 1994; Laeng, 2013) generalizes also to spacetime representations (Stocker, 2014; Stocker and Laeng, 2017) and that, if the concepts of time tend to be parasitic on the concepts (and lexicon) of space, then we should be aware and speak of time in two fundamental “modes.” In one, time flows continuously or analogically (especially within the specious present); in the other, time happens within discrete categories or regions of time events (e.g., on Tuesday, Christmas, in the 17th century; especially when conceiving the past and the future). Indeed, the path of a clock’s hands can run both in discrete steps across regions of time (e.g., consider the typical jerky movements of the seconds hand of most quartz watches) or in a continuous manner (e.g., in mechanical movement watches, the seconds’ hand rotates smoothly). As discussed by Teigen et al. (2017), when the human perceiver considers time events, these are seen as divided in sequences of distinct lifetime episodes and not so much as a continuous and seamless flow. Perhaps when participants positioned first December or March on a radial “coordinate” of the circle and, afterwards, they provided the “categorical” positions (on a hypothetical clock’s face) for the same months, each of these questions probed one of two fundamental representations of time. The former would be “coordinate” time, which is continuous, the latter “categorical” time, which is discrete, since it requests to organize time in separate (equally spaced) categories corresponding to cardinal periods of time (hours).

### Limitations

One methodological limitation of the present study is that we “primed” only one spatial form by requesting to place the month on a circle. It would have been optimal to prime other spatial forms. However, the study was exploratory (since we did not know in advance the full variety of spatial forms that respondents could entertain); in addition, there were obvious practical advantages in posing one simple question or task to the readers of the website. Another limitation is that we also queried about only two spacetime positions on the circle. Probing more months could have better revealed the underlying spatial forms and the direction of time movements. We obtained responses to the survey mainly in early December, which may have primed respondents to think of the beginning of the month instead of its end. If so, the stretched imagined time between December and March may be a result of this focus on starting points. Although we considered the age of our respondents, we have no knowledge of when specific forms arise; especially if they are present in young children and at which age they tend to appear. Finally, any of the individual characteristics we had considered as potentially modulatory factors, despite showing statistically significant effects, revealed quite small effect sizes. However, “negative” findings are only apparently a limitation, especially when they are far from being inconclusive. Given the size of the present sample, we believe that the present study provide a window onto the rather negligible impact of factors like gender, handedness or demography, on adopting a non-linear spatial models of time. In fact, null findings are just as informative and theoretically relevant as positive ones (Rosenthal, 1979; Fanelli, 2010; Munafò and Neill, 2016).

## Conclusions

People make sense of time by framing it within a spatial system; in that way, we can mentally manipulate an immaterial thing like time, as a visual or tactile object would be, in our imagery space. Our human experience in navigating and observing motion in space constitutes a useful, if not necessary, cognitive structure for thinking about time (Duffy, 2014). Although, the variety of available spatial frames (or the presence at all of a spatial frame; Bender and Beller, 2014; Sinha and Gärdenfors, 2014) reflects wide cultural and historical influences (e.g., the existence of clocks, where time “circles around”), spatializations may reflect deeper and prior psychological forces, constructed over lifetimes by individuals’ observations of the iterative nature in time of both physical and social events. No spatial model may dominate, but depending on context, some may have advantages when thinking about time. When time is conceived as a long-term process with an abrupt beginning and an irrevocable end, a mental line may work best. When thinking about temporal events as recursive or eternally returning, a ring, wheel, or circle may seem just the right form of image.

## Data Availability Statement

The datasets analyzed for this study can be found in https://nrkbeta.no/2018/01/30/lagnoefint/ (by clicking on “*ÅrFinalPublic*”).

## Ethics Statement

We conducted the whole data collection in the *NRKbeta* subsection of the general media platform NRK.no, containing information, news, and entertainment from the Norwegian Broadcasting Company (NRK), which is the Norwegian government-owned radio and television public broadcasting company, and the largest media organization in Norway. Participation in the survey was entirely voluntary and took place on the web platform of NRK. No written informed consent for participation was required for this study in accordance with the institutional requirements. Surveys conducted on the website are approved internally by the officers of the broadcasting company, following national guidelines on the handling of personal data, stating the following: “NRK’s social mission as public broadcaster gives us a special responsibility for fulfilling democratic, social and cultural needs, reflecting the diversity in society, and providing balanced case studies. NRK’s offerings should be a source of insight, reflection, experience and knowledge.” All respondents were anonymous, and their actual location, IP number, or other identifying information (e.g., name) were not registered.

## Author Contributions

AH conceived and designed the study. AH organized the database. BL performed the statistical analyses. BL wrote the first draft of the manuscript and AH edited parts of the manuscript. Both authors contributed to the manuscript revision, read and approved the submitted version.

### Conflict of Interest

AH was employed as journalist by company NRK, Norwegian Broadcasting Company, Norway.

The remaining author declares that the research was conducted in the absence of any commercial or financial relationships that could be construed as a potential conflict of interest.

## Footnotes

^1^https://nrkbeta.no/2017/11/25/dugnad-hvis-aret-er-en-sirkel-hvor-er-mars-og-desember-i-ditt-hode/

^2^https://jasp-stats.org/
